# Lack of Influence of Non-Overlapping Mutations in *BRAF*, *NRAS*, or *NF1* on 12-Month Best Objective Response and Long-Term Survival after Checkpoint Inhibitor-Based Treatment for Metastatic Melanoma

**DOI:** 10.3390/cancers15133527

**Published:** 2023-07-07

**Authors:** Alyssa Panning, Wolfram Samlowski, Gabriel Allred

**Affiliations:** 1 Kirk Kerkorian School of Medicine at UNLV, Las Vegas, NV 89106, USA; panninga@unlv.nevada.edu; 2 Comprehensive Cancer Centers of Nevada, Las Vegas, NV 89148, USA; 3 School of Medicine, University of Nevada, Reno, NV 89557, USA; 4 Gables Statistical Consulting, Bella Vista, AR 72714, USA; gabrielallred@gmail.com

**Keywords:** ipilimumab, nivolumab, pembrolizumab, dabrafenib, trametinib, encorafenib, binimetinib

## Abstract

**Simple Summary:**

Most melanoma patients have non-overlapping “driver” mutations in either BRAF, NRAS, or NF1 genes based on Next-Gen sequencing. Other overlapping genetic changes, termed “passenger mutations” may also be identified. The impact of these mutations on cancer immunotherapy outcome is currently not well understood. We evaluated the outcome of checkpoint inhibitor-based immunotherapy in 73 patients. Rare patients with BRAF fusion genes or internal rearrangements had a significantly reduced progression-free and overall survival. No other “driver” or “passenger” mutations appeared to influence outcome in a multivariate analysis. The strongest predictor of long-term survival in our study appeared to be development of a complete response as assessed at 12 months from the start of treatment.

**Abstract:**

Background: Non-overlapping somatic mutations in BRAF, NRAS, or NF1 genes occur in 85% of metastatic melanoma patients. It is not known whether these mutations affect immunotherapy outcome. Materials and methods: Next-Gen sequencing of 324 oncogenes was performed in 73 metastatic melanoma patients. A retrospective review of immunotherapy outcome was performed. Results: BRAF fusions/internal rearrangements, BRAF V600E, NRAS, NF1 mutations, and triple-negative genotypes occurred in 6.9%, 30.1%, 17.8%, 32.9%, and 12.3% of patients, respectively. Median potential follow-up was 41.0 months. Patients with BRAF fusion/rearrangement had decreased progression-free and overall survival (*p* = 0.015). The other genotypes each had similar progression-free and overall survival. Patients who achieved a complete best objective response at 12 months (*n* = 36, 49.3%) were found to have significantly improved survival compared those who failed to achieve remissions (*n* = 37, 50.7%, *p* < 0.001). Conclusions: The most important determinant of long-term survival was achievement of a complete response by 12 months following immunotherapy. PR and SD were not a stable type of response and generally resulted in progression and death from melanoma. Rare patients with BRAF fusions or rearrangements had decreased progression-free and overall survival following initial immunotherapy. Other BRAF, NRAS, or NF1 mutations were not associated with significant differences in outcome.

## 1. Introduction

Melanoma has the potential to be a highly aggressive and lethal form of cancer. The incidence of melanoma has steadily increased over the past three decades. In 2021, there were more than 100,000 new cases of melanoma diagnosed in the US and approximately 7000 of these patients died from metastatic disease [1]. Though incidence of melanoma continues to increase, the mortality rate and prognosis for melanoma has improved within the past 15 years due to advances in detection and treatment [2], including the development of effective immunotherapy and targeted therapy.

The functional basis of immunotherapy relies on the concept that many cancers, including melanoma, have evolved mechanisms to evade and exhaust the immunological response [3]. These mechanisms include the activation of T cell inhibitory receptors [4,5]. Immunotherapeutic agents that block inhibitory checkpoints such as PD-1 and CTLA-4 have shown significant activity in reactivating effective anticancer immune responses. PD-1 and CTLA-4 antibodies can be used alone as monotherapy, or together as combined dual agent therapy [6]. The development of immune checkpoint inhibitors (ICI) has led to a marked improvement in survival in metastatic melanoma patients. In contrast to a historical 1-year survival rate for metastatic cutaneous melanoma of 15% in the chemotherapy era [7], current treatment has achieved higher levels of survival. In a mature trial, a minimum of 6.5-year survival with combined ipilimumab plus nivolumab was 57%, versus 43% with single agent nivolumab and 25% with single agent ipilimumab [8].

In addition, a pattern of recurring somatic gene mutations has been identified in melanoma [9]. These mutations most commonly involve the RAS/RAF/MEK/ERK signaling pathway which mediates melanoma cell growth and survival [10]. The most common of these mutations occurs in *BRAF* at V600. Other non-overlapping mutations have been described in *NRAS* (Q61 and G12/13), as well as a diverse group of loss of function mutations in *NF1* [11,12]. *NF1* loss-of-function mutations also act to increase RAS activity [13]. The non-overlapping mutations in *BRAF*, *NRAS*, and *NF1* are sometimes referred to as “driver” mutations, due to their ability to independently promote growth, proliferation, and survival of melanoma cells [14]. Additional overlapping oncogene mutations are also frequently detected, which are sometimes termed “passenger” mutations due to their less well characterized role in melanoma growth and progression [14].

There is extensive experience with treatment of melanomas containing *BRAF* V600E somatic mutations using small molecule *BRAF* ± MEK inhibitors. Combined *BRAF* and *MEK* inhibition has resulted in prolonged progression-free and overall survival when compared to BRAF inhibitor monotherapy [15]. These agents produced a rapid onset of tumor regression and a high objective response rate. However, adaptive tumor resistance to *BRAF* and MEK inhibitors usually develops despite ongoing treatment [15]. For example, 5-year overall survival for patients treated with the *BRAF* and MEK inhibitors dabrafenib plus trametinib was 34% with a 5-year progression free survival of only 19%, despite continuous treatment [16]. Similar results were observed after long-term follow up of *BRAF* mutant melanoma treated with a different BRAF plus MEK inhibitor combination, utilizing encorafenib with binimetinib [17]. In metastatic melanoma patients who lack *BRAF* V600E mutations (including patients with *NRAS* and *NF1* mutation), targeted therapy has shown minimal effectiveness [18,19,20].

It has been suspected that the sequence of targeted therapy and immunotherapy may be an important determinant of long-term responses and survival in BRAF mutant melanoma patients. Previous retrospective patient series suggested that initial ICI-based immunotherapy produced a more favorable outcome than initial *BRAF*-directed therapy [21,22]. More recently, the DREAMseq trial (EA 6134) confirmed that initial ICI treatment with ipilimumab/nivolumab produced superior progression-free and overall survival, as well as improved response duration, compared to initial therapy with dabrafenib/trametinib in patients with *BRAF* mutant melanoma [23]. Thus, the use of initial immunotherapy is likely to become the de facto standard-of-care.

The role of the genetic background of melanoma “driver” mutations in modulating ICI responses is not yet clear. The natural history of *BRAF* and *NRAS* mutant melanoma suggests an increased risk of progression and increased frequency of metastatic disease, including an increased frequency of brain metastases [24,25,26]. Due to delays in molecular testing in a community setting, we have treated a series of genetically defined metastatic melanoma patients with initial immunotherapy. The purpose of the current retrospective analysis was to evaluate whether the mutational signature of metastatic melanoma had an impact on ICI treatment outcomes such as progression-free and overall survival.

## 2. Materials and Methods

### 2.1. Subjects and Design

Potential subjects for this retrospective chart review were identified by performing a search of Health Information Portability and Accessibility Act (HIPAA)-compliant IKnowMed medical record program (McKesson, Houston, TX, USA). This database was searched for patients with a melanoma diagnosis who had received treatment with ipilimumab, nivolumab, or pembrolizumab. This search yielded 134 potentially eligible patients treated from 2015 to 2020. We also retrieved a list of our melanoma patients who had been tested for a panel of somatic (tumor) mutations via Foundation Medicine (Cambridge, MA, USA). Overlapping patients from the two patient lists were used in the current analysis. Patients with *BRAF*, *NRAS*, *NF1* mutations, as well as those who lacked these mutations (triple negative) were identified for this study.

### 2.2. Study Methods

Patients who received initial checkpoint inhibitor therapy simultaneously with other drugs or patients who received adjuvant ICI therapy were excluded. Other exclusion criteria were presence of significant confounding illnesses occurring concurrently with melanoma, such as the presence of another malignancy. Patients who received only one dose of ICI therapy due to rapid disease progression were also excluded.

Data collected included the following: patient ID, driver mutation status (*BRAF*, *NRAS*, *NF1*, or triple negative), other concomitant somatic mutations present in tumor, as well as the ICI treatment regimen (agent, dose, start date, # of doses). The date of progression or the date of last clinic follow-up (if in remission) was recorded. ICI-induced toxicity was noted. If a subject died, the date of death and cause of death were extracted. Progression-free survival was calculated from the start of ICI therapy. If a patient required second-line therapy (e.g., targeted therapy added to ongoing PD-1 antibody therapy) to achieve a response, this was also recorded. Overall survival was calculated from the start of ICI therapy. Following completion of data extraction, patient identifying information was deleted. This study design was formally reviewed by the WCG IRB chair and was deemed exempt from full IRB review.

### 2.3. Treatment Regimens

In our practice, patients with *BRAF*, *NRAS*, or *NF1*-mutant melanomas were always treated initially with a standard ICI regimen, due to frequent delays in obtaining molecular sequencing data. These regimens included standard doses of pembrolizumab, nivolumab, or the combination of ipilimumab plus nivolumab (employing either the original regimen or using an alternate dosing regimen [27]). If patients progressed after initial ICI therapy, clinical trial participation was offered. If not eligible for clinical trials, patients with a *BRAF* mutation were treated with cautious addition of a low dose *BRAF* ± *MEK* inhibitor (typically consisted of dabrafenib 75 mg/day with or without trametinib 1 mg/day or alternatively encorafenib 75 mg/d with or without binimetinib 15 mg b.i.d.) with continuation of PD-1 antibody therapy [28]. If patients had an *NRAS* or *NF1* mutation, cautious addition of a MEK inhibitor (trametinib, binimetinib, cobimetinib) with ongoing PD-1 therapy was offered [29].

### 2.4. Response Assessment

The best objective response (BORR) was assessed at 12 months from the start of therapy using RECIST 1.1 criteria [30]. Complete response (CR) was defined as disappearance of all target and non-target lesions. Rare patients with apparent stable disease on radiographs underwent a biopsy of residual lesions to verify a pathologic complete response. Partial response (PR) was defined as more than a 30% reduction in sum of bidimensional tumor measurements. Progressive disease (PD) was described as >20% increase in sum of bidimensional tumor measurements or the development of new metastases. Stable disease (SD) was defined as any response not meeting criteria for CR, PR, or PD. Data collection concluded 1 August 2022 (with a minimum potential follow-up of 18 months).

### 2.5. Statistical Analysis

Data were entered into an Excel spreadsheet (Microsoft, Redmond, WA, USA). Patients were divided into groups based on driver mutation status (*BRAF*, *NRAS*, *NF1*, or triple wild type). Descriptive statistics, such as median, standard deviation, and data range were calculated. Overall survival (OS) and progression-free survival (PFS) were analyzed for each mutation group using methods described by Kaplan and Meier [31]. Comparison between mutation groups was performed using Chi-squared analysis. Analysis of progression-free and overall survival by best objective response at 12 months was also performed. This included patients that were converted from progressive or stable disease to complete remission with the addition of targeted therapy (dabrafenib, vemurafenib, encorafenib, trametinib, cobimetinib, or binimetinib) to PD-1 antibody treatment. Comparison of survival curves was performed via Log-rank test [32,33]. Independent sample *t*-tests were conducted comparing differences in overall survival (OS) and progression free survival (PFS) between driver mutation status groups. Additionally, independent sample *t*-tests were conducted examining OS and PFS outcomes between passenger mutations. Differences in OS and PFS were analyzed using one-way analysis of variance (ANOVA) between BORR groups. Additional pairwise comparisons between BORR groups were made using a Bonferroni adjustment for multiple comparisons.

## 3. Results

### 3.1. Patient Characteristics

We identified a total of 73 patients who had both Foundation Medicine CDx testing of their tumor tissue and were treated with initial ICI therapy for metastatic melanoma. This included anti-PD-1 antibodies, either alone (*n* = 27) or in combination with anti-CTLA-4 antibodies (*n* = 46). Patient characteristics are provided (Supplementary Tables S1 and S2). The median age of patients at the start of treatment was 63.3 years. Median duration of potential follow-up was 41.0 months (range 19.6–128.2 months).

A total of 60.3% of patients developed disease progression on initial ICI therapy, and therefore required second line treatment. Of those who progressed, 61.3% received targeted therapy such as *BRAF* or *MEK* inhibitors, in combination with continued *PD-1* antibody treatment [28,29]. Other second line treatments included clinical trial participation (9.1), radiotherapy (22.7%), other tyrosine kinase inhibitors (4.5%), chemotherapy (6.8%), and talimogene laherparevec (TVEC) (4.5%). Some patients received treatment with more than one therapeutic modality.

At the end of data collection, 13.7% of the patients were still receiving ongoing treatment, 43.8% were alive and had discontinued all treatment after achieving confirmed complete response [34], and 41.1% were deceased. One patient (1.4%) died of an unrelated illness. Of the patients who were deceased, the majority (96.7%) died from progressive metastatic melanoma despite ongoing therapy.

### 3.2. Mutation Frequency

**Figure 1:**
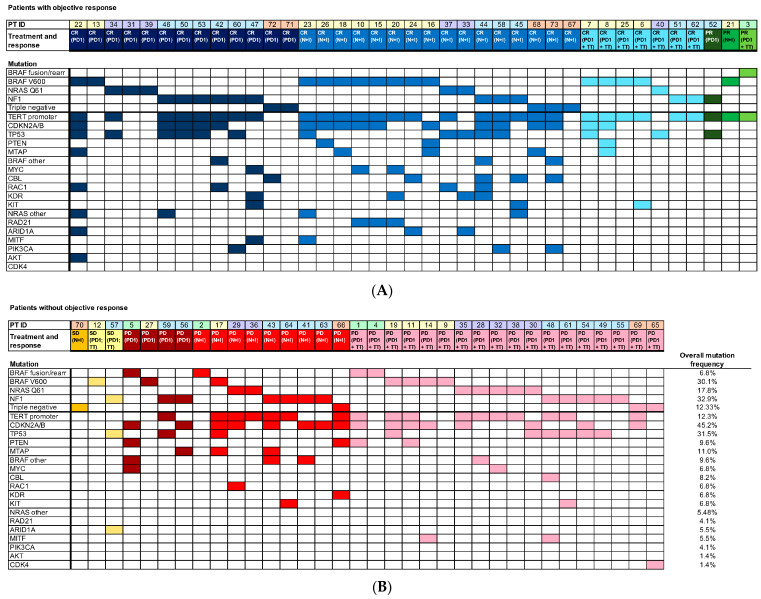
Patient genotype and treatment response. The spectrum of oncogene mutations in metastatic melanoma patients grouped by treatment response to ICI therapy (single agent PD-1, dual CTLA4 plus PD-1 therapy, and targeted therapy added to ongoing PD-1 directed therapy). (**A**) CR or PR as BORR at 12 months; (**B**) SD or PD as BORR at 12 months.

Patients were analyzed according to “driver” mutation status (Figure 1): 6.9% had a *BRAF* fusion/rearrangement mutation, 30.1% had a *BRAF* V600 mutation, 17.8% had a mutation in *NRAS*, 32.9% had an *NF1* mutation, and the remaining 12.3% were wild type for *BRAF*, *NRAS*, and *NF1* (as well as *C-KIT*). Of the 22 *BRAF* V600 mutant patients, most had a V600E mutation (72.7%), while a small number had a V600K or V600R mutation (22.7% and 4.5%, respectively). The most common mutations in the *NRAS* gene were Q61R (53.8%), Q61K (30.8%), and Q61L (15.4%). A wide spectrum of *NF1*-inactivating mutations was observed. Overlapping “passenger” mutations were identified in additional oncogenes (Figure 1). The most common were *TERT* promoter gene deletions (64.9% of patients), or in *CDKN2A/B* (44.6%), and *TP53* (31.1%). Median tumor mutational burden (TMB) was 15.0, with a high of 155 and a low of 0. The pattern of responses in relation to tumor genotype is also shown (Figure 1). Of the patients who achieved a CR with treatment, 80.6% achieved a CR following initial ICI therapy, and 19.4% required 2nd line treatment with *BRAF* and/or *MEK* inhibitors with continuation of PD-1 antibody treatment (Figure 1).

### 3.3. Analysis of Treatment Outcome

**Figure 2:**
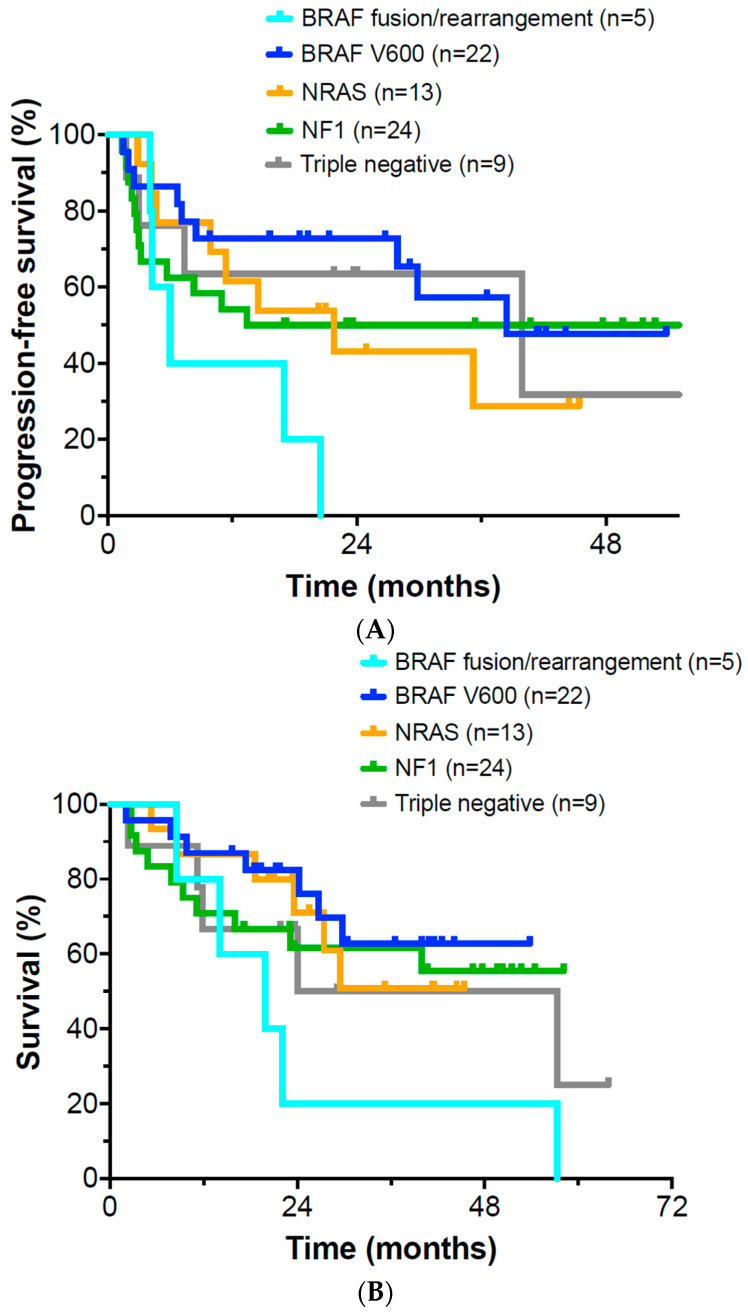
(**A**) Progression-free survival in patients with *BRAF*, *NRAS*, and *NF1* and “triple negative” mutations; (**B**) Overall survival in patients with *BRAF*, *NRAS*, and *NF1* and “triple negative” mutations.

Median potential follow-up of patients was 41.0 ± 21.2 months. Median progression free survival was 23.6 months overall in the entire cohort. Patients with *BRAF* fusions or gene rearrangement had a median PFS of only 6.0 months (Figure 2A). Patients with *BRAF* V600, *NRAS*, *NF1* mutations, and “triple negative” patients had a median PFS of 24.0, 20.3, 15.2, and 21.8 months, respectively.

Median overall survival (OS) was 23.6 months for the entire group of patients. Median OS for patients with BRAF fusion/rearrangements was 19.9 months (Figure 2B). OS of patients with mutations in BRAF V600E, NRAS, and NF1 was 25.4, 25.5, and 23.1 months, respectively. OS of “triple negative” patients was found to be 18.2 months. Of the BRAF V600 patients who initially progressed on immunotherapy, 28.6% were converted to a complete remission after the addition of targeted therapy to ongoing PD-1 Mab administration, as were 16.7% of patients with an NRAS mutation and 18.2% with an NF1 mutation. No triple negative patients received targeted therapy. Univariate *t*-tests (Table 1) and one-way analysis of variance (Table 2 and Table 3) were performed to evaluate the potential association of somatic mutations with PFS and OS. Patients with BRAF fusions and rearrangements had significantly reduced PFS and OS. In contrast, there was no statistical association between mutation status and overall survival in log rank analysis when evaluating the other mutation groups (*p* = 0.19). In exploratory analyses, there was also no observed correlation between initial lactate dehydrogenase (LDH), Karnofsky performance scale rating, or tumor mutation burden (TMB) and overall survival. In univariate analysis, “passenger mutation” status was associated with increased PFS only for the RAC1 (*n* = 5, 6.8%, *p* = 0.027), CBL (*n* = 6, 8.2%, *p* = 0.087), and KDR (*n* = 5, 6.8%, *p* = 0.099) mutations. None of these “passenger mutations” proved significant in multivariate analysis. Our multivariate analysis suggested that the best predictor of PFS or OS was a complete remission at 12 months.

We confirmed that a complete response as the BORR at 12 months was predictive of progression-free and overall survival. Overall, the BORR at 12 months was a complete response (CR) in 49.3% of patients, while 50.7% had PR, SD, or PD as their best response. The percentage of patients who achieved CR as the BORR after initial ICI treatment was 45.5% for the *BRAF* V600 mutation subset, 38.5% for *NRAS* mutant patients, 37.5% for *NF1* mutant patients, and 55.6% for triple negative patients (*p* > 0.05 via chi-square test). No patients with a *BRAF* fusion or gene rearrangement responded to ICI or 2nd line PD-1 antibody plus targeted therapy treatment (BORR of 0%). Of the 50.7% group that did not achieve a CR, only a small number of patients had a BORR of stable disease (SD) or partial response (PR) (4.1% each). Virtually all these patients eventually developed progressive disease (PD) and died.

**Figure 3:**
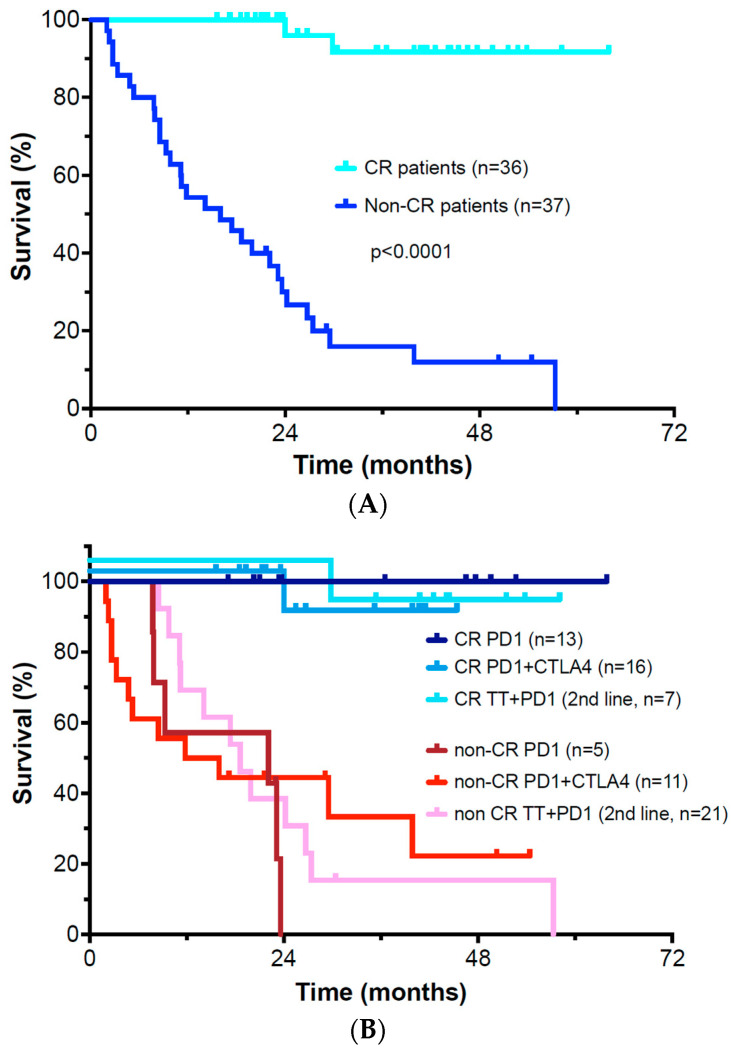
(**A**) Overall survival in patients achieving a complete response versus less than complete response as best objective response; (**B**) Overall survival of patients achieving CR versus non-CR patients grouped by first line therapy (single agent PD-1 versus ipilimumab plus nivolumab) or second line therapy (cautious addition of targeted therapy to ongoing PD-1 monotherapy).

The overall survival outcome of ICI-based therapy appeared to be dichotomous based on BORR response assessment (Figure 3A). Patients who achieved a BORR of complete response with either 1st or 2nd line immunotherapy-based treatment (*n* = 36, 49.3% of patients) had a significantly better survival than patients whose response was PR, SD, or PD (*n* = 37, 50.7% of patients) (*p* < 0.0001). PR and SD were not a stable type of response. Only 6 patients (8.2%) had a BORR at 12 months of partial response or stable disease. Those 6 patients were included in the non-CR group for analysis, as most eventually progressed and died of disease (2 currently continue to receive treatment). Patients who were converted to a CR with 2nd line continuation of PD-1 antibody treatment with addition of TT also appeared to achieve improved long-term survival (Figure 3B) (*p* < 0.0001).

## 4. Discussion

It is currently suspected that the sequence of targeted therapy and immunotherapy may be an important determinant for durable long-term responses and survival. It had been suspected that initial immunotherapy followed by targeted therapy has a better outcome than the converse [21,22]. The recent DREAMseq Phase III trial (Alliance trial EA6134) randomized *BRAF*-mutant patients to either initial ipilimumab/nivolumab immunotherapy or initial targeted therapy with dabrafenib/trametinib. Patients subsequently underwent a planned crossover at progression to the alternate regimen. This study was closed at interim analysis, since the 2-year overall survival rate for *BRAF* V600 melanoma patients treated with ICI first was 72%, while the reverse treatment sequence (targeted therapy first) had a 2-year overall survival of only 52% [23]. Based on this trial, the use of initial immunotherapy is likely to become standard-of-care.

The role of the genetic background of melanoma “driver” mutations in modulating ICI responses is not yet well understood. There has been a suspicion that the natural history of *BRAF* and *NRAS* mutant melanoma is more aggressive than melanomas lacking these mutations [25,35,36].

We identified two prior studies of the effect of common non-overlapping melanoma mutations on treatment outcome. Van Not et al. published the outcome of ICI therapy in a large multi-institutional series of patients who underwent genotyping via a variety of techniques to specifically identify BRAF and NRAS mutations [37]. Most of these patients were subsequently treated with CTLA4 plus PD-1 antibodies or PD-1 monotherapy. These investigators concluded that patients with BRAF mutations had superior PFS and OS compared to NRAS treated patients, especially following combined therapy. Another single institution study by Möller et al. evaluated the outcome of patients who were identified to have c-KIT or NRAS mutations [38]. BRAF mutant patients were excluded from the analysis. The testing methodology was not described. Patients with “wild type” genetics appeared to have superior survival to NRAS mutant patients, particularly if treated with ICI, as compared to chemotherapy. NF1 mutation status was not characterized in “wild type” patients. Neither study identified BRAF fusions or rearrangements.

We have extended these prior results by evaluating all currently testable non-overlapping somatic oncogene mutations in cutaneous melanoma. The mutation spectrum was assayed simultaneously in all patients using a single well validated Next-Gen sequencing platform. It should be noted we excluded acral lentiginous and mucosal melanoma, thus there were no c-KIT mutations identified. C-KIT mutations also would be expected to be non-overlapping with other “driver” mutations. All patients were treated in a uniform fashion by a single investigator. Our patient series found that BRAF fusion and rearrangement patients had a significantly inferior outcome. Outcomes in other genetic subsets, such as BRAF V600 mutations, NRAS, NF1, and “triple negative” patients appeared to have similar treatment outcomes, in contrast to other published studies [37,38]. Univariate and multivariate analysis suggested that the best objective response, assessed at 1 year, was highly correlated with long term survival. Patients who achieved a BORR of complete response with either 1st line or 2nd line ICI treatment (with addition of targeted agents in second line) appeared to have superior long-term survival compared to patients with BORR of PR, SD, or PD. This implies efforts to achieve a complete remission are important in melanoma outcome. The usefulness of BORR as a study endpoint will require further validation in prospective trials.

In our patient series, 5 of 13 (38.5%) patients with *NRAS* mutations achieved a complete remission with ICI treatment. One additional NRAS mutant patient was converted to CR with the addition of *MEK* inhibitors to PD-1 maintenance therapy (7.7%). In patients with *NF1* mutations, 9 of 24 (37.5%) achieved a complete remission with initial immunotherapy. Two more patients were converted to CR by addition of *MEK* inhibitors to ongoing PD-1 therapy (8.3%). In “triple negative” patients, 5 of 9 (55.6%) achieved a CR with initial immunotherapy. None of the progressing “triple negative” patients were able to be salvaged by addition of targeted therapy. The “triple negative” subset of patients requires further evaluation in clinical trials to define appropriate salvage treatment options. In our series, *BRAF* V600 patients who progressed on initial ICI therapy also had substantial rates of conversion to complete response (28.6% overall) with the cautious addition of *BRAF* ± *MEK* inhibitors to ongoing PD-1 directed therapy [28]. Using this approach, another 4 out of 9 *BRAF* V600 patients who progressed after initial immunotherapy were converted to a durable complete response.

We observed that patients with a *BRAF* fusions or rearrangements did particularly poorly: all 5 of these patients progressed and died despite treatment with immunotherapy and subsequent 2nd line addition of TT. *BRAF* fusion/rearrangement status was associated with decreased median PFS relative to the overall study population. Our data suggest that neither immunotherapy nor targeted therapy had a durable benefit in this group of patients. Thus, development of more effective treatment approaches for this adverse prognostic group is badly needed. It should be noted that these mutations were only identified by the Next-Gen sequencing panel and would have been missed by BRAF V600E specific testing.

Historically, response assessment such as RECIST criteria was developed to evaluate clinical responses to chemotherapy [30]. Since these responses are often transient, response assessment was usually performed after only 2–3 months of treatment. When applied to immunotherapy, these response criteria have been less than optimal, as immunotherapy responses are slow to develop and often continue to evolve over many months. This has led to alternate response assessment tools, such as the Immune RECIST criteria [39]. We chose the best overall response rate (BORR assessed at 12 months post-treatment) as a useful outcome assessment tool.

We were surprised to find a strongly dichotomous BORR response pattern to treatment in both univariate and multivariate statistical analysis. Almost half of our patients responded to 1st or 2nd line therapy with a CR over a 12-month span. Patients who achieved a complete response had an improved overall survival (median undefined in CR patients vs. 16.0 months for non-CR patients, *p* < 0.001). At a median follow-up of 41 months, 91% of CR patients were alive and disease free and most had discontinued therapy as we have previously described [34]. It is notable that patients who achieved a CR with second line PD-1 + TT therapy appeared to have a similar survival benefit to patients who achieved a CR following initial immunotherapy. Virtually all patients with less than a CR have progressed and died of their disease. Thus, PR and SD appeared to be transient and unstable responses. Only 6 patients had a BORR of PR or SD, and all eventually progressed. Thus, an important conclusion from our study is that increasing the percentage of complete responses following 1st or 2nd-line checkpoint inhibitor-based therapy seems important to achieving improved outcomes.

There are potential caveats to our conclusions. All patients underwent uniform Next-Gen-based oncogene sequencing. It should be noted that some insurance companies deny this important testing. Whether inadvertent patient selection bias is introduced by these denials is unclear. It also remains to be established whether our conclusions can be applied to other molecular genetic testing platforms. It is possible that differences in immunotherapy response between the different genetic subsets were not detectable due to our relatively small sample size. We are, therefore, planning to confirm our preliminary data in a larger multi-institutional analysis. In addition, this publication represents patients treated over a 5-year span with several different ICI regimens, based on the timing of regulatory approvals. Prospective evaluation of our findings in a uniformly treated patient cohort is needed. Thus, the current study is meant to be hypothesis generating. We believe our data may inform potential future studies on the molecular factors influencing treatment response as well as attempts to increase the complete response rate in metastatic melanoma.

## 5. Conclusions

The use of initial immunotherapy for metastatic melanoma is likely to become standard-of-care, prior to the use of targeted therapy. *BRAF*, *NRAS*, and *NF1* mutational status in metastatic melanoma was not statistically associated with differences in either overall survival or progression-free survival, except for rare patients with *BRAF* fusions or internal *BRAF* gene rearrangements. These patients have an exceptionally poor outcome with contemporary ICI and TT therapy and require the development of more effective treatment options. Based on our data, it is possible that some somatic “passenger” mutations or gene amplifications identified in tumor biopsies may further modulate treatment responses. These did not reach statistical significance in our dataset due to small patient numbers. This will need further evaluation in a larger multi-institutional cohort of patients. The most significant determinant of long-term survival appeared to be the ability to produce a complete remission. A better understanding of the biologic factors that mediate ICI-induced complete responses are still needed, as many of the currently known prognostic factors did not seem to statistically correlate with outcome.

## Figures and Tables

**Table 1:** Independent samples *t*-test analysis of mutations.

		Overall Survival in Days (OS)						Progression-Free Survival in Days (PFS)					
Driver Mutation	N	Mean	SD	Median	Min	Max	*p*	Mean	SD	Median	Min	Max	*p*
All Patients	73	818.9	487.38	720	61	1950	--	633.1	521.31	524	43	1950	--
BRAF fusion/rearr.	5	744	584.6	606	259	1749	0.724	316.2	237.14	182	125	625	0.161
BRAF V600	22	841	414	776	61	1642	0.801	726.5	483.97	731.5	46	1642	0.318
NF1	13	820.15	309.9	778	258	1386	0.992	611.15	439	620	87	1386	0.869
NRAS	24	831.25	606.37	704	82	1772	0.895	624.42	596.76	464	43	1772	0.922
“Triple negative”	9	771.78	555.03	722	69	1950	0.759	635.33	634.25	665	54	1950	0.989
CDKN2AB	33	843.7	454.57	732	61	1749	0.696	636.53	485.63	524	46	1570	0.951
TERT promoter	47	862.6	491.49	732	61	1772	0.306	684.28	520.02	589	43	1772	0.262
PTEN	7	812.43	500.99	650	360	1749	0.971	504.71	353.97	477	182	1170	0.497
TP53	23	851.17	521.43	778	61	1570	0.704	683.17	545.98	524	43	1570	0.581
MTAP	8	625.5	426.69	647	61	1245	0.237	536.75	451.48	533	61	1170	0.583
BRAF passenger	7	728	507.38	675	83	1455	0.607	655.14	531.6	625	61	1455	0.907
MYC	5	925	466.91	675	563	1606	0.615	762	545.1	625	128	1606	0.570
CBL	6	986.5	642.02	981	82	1950	0.383	982.17	649.39	981	56	1950	0.087
RAC1	5	1132.4	247.69	1112	778	1455	0.137	1128.4	254.9	1112	758	1455	0.027
KDR	5	1031.4	540.93	1242	360	1606	0.316	1004.4	584.44	1242	225	1606	0.099
KIT	5	999.2	650.88	909	102	1659	0.395	671.6	647.48	720	43	1606	0.865
NRAS passenger	6	856	467.73	916	161	1353	0.847	845.33	487.08	916	97	1353	0.301
RAD21	3	864.67	330.54	813	563	1218	0.869	754.67	170.17	813	563	888	0.683
ARID1A	4	1115.25	296.77	1186	703	1386	0.213	1115.25	296.77	1186	703	1386	0.056
MITF	4	923.5	664.82	1003	82	1606	0.662	796.75	756.08	762.5	56	1606	0.522

Abbreviations: N, number; SD, standard deviation; Min, minimum; Max, maximum; green—statistically significant (*p* < 0.05); yellow—approaching statistical significance.

**Table 2:** BORR descriptive statistics.

		Overall Survival in Days (OS)						Progression-Free Survival in Days (PFS)			
Response Type	N	Mean	SD	Median	Min	Max	Mean	SD	Median	Min	Max
Complete response	36	1062.6	411.3	1095.5	447	1950	1002.2	443.9	898.5	93	1950
Partial response	3	517.6	204.3	606	284	663	356.6	141.4	298	254	518
Stable disease	3	566.3	445.0	703	69	927	537.3	420.7	703	59	850
Progressive disease	31	589.4	464.2	489	61	1749	240.3	278.8	131	43	1218

Abbreviations: N, number; SD, standard deviation; Min, minimum; Max, maximum.

**Table 3:** Pairwise comparisons between BORR via one-way ANOVA with Bonferroni adjustment for multiple comparisons.

		Overall Survival in Days (OS)			Progression-Free Survival in Days (PFS)		
BORR	Comparator	Mean Difference	Std. Error	*p*	Mean Difference	Std. Error	*p*
CR	PR	545	259.5	0.236	645.556	224.427	0.032
	SD	496.333	259.5	0.36	464.889	224.427	0.252
	PD	473.247	105.809	<0.001	761.867	105.809	<0.001
PR	SD	−48.667	352.591	1.000	−180.667	352.591	1.000
	PD	−71.753	261.105	1.000	116.312	261.105	1.000
SD	PD	−23.086	261.105	1.000	−296.978	225.815	1.000

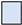
 Statistically significant (*p* < 0.0083), All genetic mutations with a *p* value approaching statistical significance were included in the multivariate ANOVA analysis, but none reached significance.

## Data Availability

De-identified primary data will be made available upon reasonable request to the corresponding author.

## Supplementary Materials

The following supporting information can be downloaded at: https://www.mdpi.com/article/10.3390/cancers15133527/s1, Table S1: Patient demographics, Table S2: Treatment characteristics and patient outcome.

## Abbreviations

ICI: immune checkpoint inhibitors; TT: targeted therapy; LDH: lactate dehydrogenase; MSI: microsatellite instability; TMB: tumor mutational burden; FFPE: formalin fixed, paraffin embedded; IRB: institutional review board; BORR: best objective response rate (at 12 months); CR: complete response; PR: partial response; SD: stable disease; PD: progressive disease; OS: overall survival; PFS: progression-free survival; RECIST: Response Evaluation Criteria in Solid Tumors.
