# Supplementary material for: Lack of Influence of Non-Overlapping Mutations in BRAF, NRAS, or NF1 on 12-Month Best Objective Response and Long-Term Survival after Checkpoint Inhibitor-Based Treatment for Metastatic Melanoma
Source: Cancers (Basel). 2023 Jul 7;15(13):3527. doi: 10.3390/cancers15133527 (PMC10340344; doi:10.3390/cancers15133527)
Supplement: Supplementary file 1 [file cancers-15-03527-s001.zip › Alyssa Supplemental Table S1 pt demographics 8-14-22.pdf]

Table 1: Patient demographics

Pt	Mut	Age	Sex	Race	Primary site	Site of metastases	Initial Stage	Initial LDH	TMB (/Mb)	PDL1 (%)	Brain mets	KPS (%)	Comorbidities
1	BRAF NF2 fusion	53	F	C	trunk	lung, SQ, adrenal, bone	IVC	115	6		N	100	HCL, obesity
2	BRAF CCDC127 fusion	65	M	C	trunk	liver, spleen, bone, LN, lung	IV	190	n/a		N	100	HTN
3	BRAF rearr exon 8	68	F	C	head	LN, abdomen, bone	IIIC	190	5	80	N	90	N/A
4	BRAF T599_V600 insT	55	M	C	leg	LN, lung, bone, brain	IIID	172	n/a		Y	100	BPH
5	BRAF del exon5-intron 7	66	M	C	leg	LN, bone, lung, liver	IV	162	5	1	N	90	back pain, HTN, renal cell carcinoma (in remission)
6	BRAF V600E	34	M	C	neck	LN, SQ, liver, spleen, lung, brain	IIIC	214	24		Y	100	GERD
7	BRAF V600E	26	M	C	trunk	adrenal, bone, axilla	III	161	5		N	100	N/A
8	BRAF V600E	51	F	C	arm	LN	IIIC	161	10		N	90	N/A
9	BRAF V600E	53	M	C	trunk	LN, brain, bone	IV	286	8		Y	80	HTN, chronic back pain
10	BRAF V600E	59	M	C	trunk	lung, bone	IIIB	248	3	0	N	90	arthritis, back pain, HCL, hypogonadism
11	BRAF V600E	43	F	C	trunk	lung, adrenal, brain, retroperitoneal	IIIB	213	6	0	Y	100	back pain, anxiety, depression
12	BRAF V600E	48	F	C	arm	SQ	IVA	175	3		N	100	back pain
13	BRAF V600E	51	F	C	leg	LN, lung	IVB	233	Ind		N	90	back pain, depression
14	BRAF V600E	59	M	C	trunk	LN, abdomen, axilla	IIIC	126	n/a		N	80	N/A
15	BRAF V600E	48	M	C	leg	SQ	IIIC	184	8		N	100	HTN
16	BRAF V600E	56	M	C	trunk	SQ, peripancreatic, bone, LN	III	215	9	5	N	90	T2D, angina, HTN
17	BRAF V600E	87	M	C	unk	SQ, liver, bone	IVA	359	14	100	N	90	T2D, aortic stenosis, CAD, HTN
18	BRAF V600E	66	F	C	leg	SQ	IIIB	189	8	10	N	100	N/A
19	BRAF V600E	36	M	C	head	SQ, LN, parotid gland	IVB	165	1	10	N	100	N/A
20	BRAF V600E	63	F	C	trunk	stomach, colon, lung	IVB	172	11	1	N	100	N/A
21	BRAF V600E	35	F	C	trunk	breast	IIB	178	14	0	N	90	N/a
22	BRAF V600E	75	M	C	head	SQ, LN	IIIC	170	44	0	N	90	HTN, hyperlipidemia
23	BRAF V600E	68	M	C	trunk	lung, muscle, LN	IVC	201	50		N	100	Afib, HTN
24	BRAF V600E	55	M	C	head	LN, SQ, lung	IVB	168	32		N	80	T2D, HTN, renal failure
25	BRAF V600E	49	M	H	head	SQ, bone	IVA	158	25		N	100	N/A
26	BRAF V600E	52	F	C	trunk	LN, brain, lung	IIIC	160	19	1	Y	90	osteoporosis, HCL, bradycardia, anxiety, depression
27	BRAF V600E	62	F	C	arm	LN, brain	IVD	276	11	1	Y	80	n/a
28	NRAS Q61R	51	M	C	trunk	LN, SQ, lung, bone	IIID	111	8		N	90	n/a
29	NRAS Q61K	49	M	C	leg	SQ, LN	IIIC	191	57	1	N	100	HTN, BPH, hypotestosteronism
30	NRAS Q61K	32	M	C	head	LN, SQ, parotid, liver, brain	IVD	167	13		Y	90	n/a
31	NRAS Q61K	79	M	C	unk	LN	IVA	423	18	0	N	90	arthritis, chronic back pain, HTN
32	NRAS Q61K	69	M	C	leg	LN, SQ, liver, lungs	IIIB	180	4	1	N	90	SCC, CAD, T2D, HTN
33	NRAS Q61L	57	M	C	unk	LN, chest wall	IVA	242	32		N	90	fatty liver, T2D, HTN
34	NRAS Q61L	66	M	C	arm	SQ	IVA	133	39	1	N	100	GERD, HCL
35	NRAS Q61R	48	F	C	arm	lung, LN, IM, SQ, bone, kidney, brain	IVB	100	n/a		Y	100	n/a
36	NRAS Q61R	63	F	C	back	LN, liver, retroperitoneum, lung, brain	IIIC	207	11		Y	100	GERD
37	NRAS Q61R	65	M	C	trunk	LN, colon	IVB	184	15		N	90	Depression, arthritis
38	NRAS Q61R	67	M	C	trunk	LN	IIIA	137	21		N	80	CAD, obesity, T2D, CHF
39	NRAS Q61R	71	M	C	arm	LN, liver, spleen, lung	IIIB	141	Ind	50	N	80	multiple sclerosis, CAD
40	NRAS T50I, G12D	72	M	C	trunk	LN, liver, lung, bone	IIIB	223	81		N	100	HTN, CAD, HCL
41	R1362	59	M	C	trunk	lung, LN	IV	120	n/a		N	80	n/a
42	NF1 E1734fs*4, R440*	100	F	C	neck	parotid, LN, pleura	IVB	220	34		N	80	afib, dementia, HTN
43	NF1 Q1174*, E1790*	77	M	C	head	brain, lung	IVD	371	82	0	Y	80	T2D
44	NF1 E1206*, Q1806*	48	M	C	head	lung, LN	IIB	135	139		N	100	HCL, HTN
45	NF1 R135W, L62*	77	M	C	unk	pancreas, liver, bone, LN	IVC	281	106	40	N	90	Hep B, HTN, GERD, anemia, thrombocytopenia, arrhythmia, CAD

46	NF1 splice site 5296-1G>A, splice site 6819+1G>A	61	M	C	head	LN, SQ	IIC	182	144	0	N	90	n/a
47	NF1 B192*	75	M	C	head	lung, liver, LN, bone, brain	IIB	146	47	0	Y	90	arthritis, HTN, gout, T2D, hepatitis
48	NF1 C1367*, T257fs*48	67	M	C	trunk	brain, liver, lung	IIA	312	79	60	Y	100	n/a
49	NF1 E547*, R1276Q	99	M	C	head	LN, SQ, lungs, bone	IIIB	164	6		N	90	arthritis, back pain, emphysema, HTN, CKD, CAD
50	NF1 E725fs*3, Q948*	75	F	C	unk	lung	IVB	409	155		N	90	HTN, HCL, CHF, Afib, COPD, anxiety
51	NF1 G1758fs*6	69	M	C	arm	LN, chest	IIA	200	54		N	100	seizure disorder, HTN, hypothyroidism, T2D, HCL, arthritis
52	NF1 K1704*	72	M	C	head	SQ	IIIC	145	34	0	N	80	T2D, HTN, gout
53	NF1 L1906fs*13	71	M	C	head	LN	IIIA	166	73		N	100	recurrent DVT, HTN
54	NF1 loss	48	M	C	head	LN, liver, SQ, conjunctiva, lung	unkn	218	5		N	100	HTN, GERD
55	NF1 loss	82	M	C	face	LN, bone, lung, sinus cavity	IVB	135	6		N	90	CAD, HTN
56	NF1 loss	53	F	C	foot	LN	IIIB	268	0	5	N	100	Stage II colon cancer (in remission)
57	NF1 Q1055*	84	M	C	scalp	lung	IIIB	136	78	99	N	90	DVT
58	NF1 Q1174*, R440*	79	M	C	scalp	SQ, LN, lung	IIA	238	154	0	N	100	HTN, hyperlipidemia
59	NF1 Q1341*, R1362*, splice site 3113+1G>A	69	M	C	unk	brain, lung, intramuscular	IVD	236	n/a		Y	70	HTN
60	NF1 R440*	81	M	C	trunk	LN, lung, SQ	IV	166	52		N	80	COPD, arthritis
61	NF1 S155fs*7	77	F	C	unk	urethra, vagina	IVC	170	1		N	100	back pain, HTN
62	NF1 S168*	77	M	C	face	SQ, adrenal, lung, LN	unk	255	82		N	90	CAD, arthritis, anxiety
63	NF1 truncation exon 10	62	F	C	trunk	bone	IVC	170	n/a		N	90	depression
64	NF-1 Y489fs*1	63	M	C		arm, lung, LN, bone	IVC	182	49		N	90	Colitis, peripheral neuropathy
65	TN	56	M	H	leg	LN, colon, liver, bone, lung	IIIB	194	2		N	100	HTN
66	TN	63	M	C	scalp	LN, chest, bone, thigh	unk	152	16	0	N	100	n/a
67	TN	63	M	C	trunk	LN, SQ, mesentery, peritoneum, scrotum	IIC	133	4	0	N	100	anxiety
68	TN	73	F	C	leg	LN, SQ, lung	IV	202	24	2	N	90	n/a
69	TN	55	M	C	leg	LN, bone	IIIA	197	11	0	N	100	HTN, back pain, arthritis
70	TN	60	M	C	trunk	lung	IVB	202	Ind		N	90	T2D, HTN, gout, HCL
71	TN	73	F	C	scalp	cutaneous	III	210	Ind		N	100	T2D, hypothyroidism, HCL, BCC
72	TN	42	M	C	foot	cutaneous, SQ, bone, IM, LN	IVA	185	1		N	90	obesity, depression, hypothyroidism
73	TN	70	M	C	scalp and trunk	lung	IIB	155	88		N	100	multiple prior skin cancers, HCL, BPH

UPN, unique patient number; TN, triple negative (no BRAF, NRAS or NF1 mutations identified); F, female; M, male; C, Caucasian; H, Hispanic; n/a, results not available; Y, yes; N, no; primary site; SQ, subcutaneous; LN, lymph node; ABD, abdomen; IM, intramuscular; unk, unknown; Ind, indeterminate; HCL, hypercholesterolemia; HTN, hypertension; T2D, Type 2 diabetes; CAD, coronary artery disease; BPH, benign prostatic hyperplasia; GERD, gastroesophageal reflux disease; COPD, chronic obstructive pulmonary disease; DVT, deep vein thrombosis; N/A, none.
