# Supplementary material for: Lack of Influence of Non-Overlapping Mutations in BRAF, NRAS, or NF1 on 12-Month Best Objective Response and Long-Term Survival after Checkpoint Inhibitor-Based Treatment for Metastatic Melanoma
Source: Cancers (Basel). 2023 Jul 7;15(13):3527. doi: 10.3390/cancers15133527 (PMC10340344; doi:10.3390/cancers15133527)
Supplement: Supplementary file 1 [file cancers-15-03527-s001.zip › Alyssa Supplemental Table S2 treatment 8-15-22.pdf]

Table 2 Treatment characteristics and patient outcomes

UPN	Mutation	CKI Regimen	CKI doses	ORR	PFS (mo)	TT added	OS (mo)	CKI toxicity	Current status
1	BRAF F/R	I/N	50	PD	6.0	D+T	57.3	hypothyroidism	DOD
2	BRAF F/R	I/N	12	PD	4.1	-	8.5	colitis	DOD
3	BRAF F/R	I/N	17	PR	17.0	D+T	19.9	–	DOD
4	BRAF F/R	I/N	13	PD	4.3	D+T; E+B	14.1	immune nephritis	DOD
5	BRAF F/R	P	8	PD	20.5	-	22.1	hypothyroidism	DOD
6	BRAF V600E	N	13	CR	29.8	E+B	29.8	–	DOD
7	BRAF V600E	I/N	13	CR (TT)	44.1	E	44.1	Fever, diarrhea	NED
8	BRAF V600E	N	8	CR (TT)	38.4	D+T; E+B	40.8	skin rash	NED
9	BRAF V600E	I/N	9	PD	2.5	E+B; E+T	9.8	–	DOD
10	BRAF V600E	I/N	16	CR	29.1	-	39.9	–	NED
11	BRAF V600E	I/N	11	PD	6.7	E+B	17.4	fevers	DOD
12	BRAF V600E	N	12	SD	27.9	D+T	30.4	hypothyroid	NED
13	BRAF V600E	N	9	CR	42.2	-	42.5	hand rash, hypothyroid	NED
14	BRAF V600E	I/N	10	PD	8.5	V; D	24.2	colitis	DOD
15	BRAF V600E	I/N	4	CR	26.7	-	26.7	hypopituitarism	NED
16	BRAF V600E	I/N	8	CR	15.6	-	15.6	probable CIDP	NED
17	BRAF V600E	I/N	3	PD	2.0	-	2.0	–	DOD
18	BRAF V600E	I/N	8	CR	19.3	-	19.3	rash, elevated LFTs, hypothyroid	NED
19	BRAF V600E	I/N	5	PD	1.5	E+B	21.6	–	PD
20	BRAF V600E	I/N	4	CR	18.5	-	18.5	encephalopathy, hypothyroid, rash	NED
21	BRAF V600E	I/N	2	PR	9.8	-	21.7	colitis, pneumonitis	NED
22	BRAF V600K	N	12	CR	36.5	-	36.5	–	NED
23	BRAF V600K	I/N	7	CR	41.5	-	41.5	hypopituitarism, skin rash	NED
24	BRAF V600K	I/N	16	CR	41.3	-	41.3	hypopituitarism	NED
25	BRAF V600K	I/N	32	CR (TT)	53.8	D+T	53.8	inflammatory arthritis; hypothyroid	NED
26	BRAF V600K	I/N	10	CR	21.3	-	21.3	hypothyroid	NED
27	BRAF V600R	N	7	PD	7.1	-	7.8	pneumonitis	PD
28	NRAS Q61R	I/N	24	PD	14.5	T; B	26.7	colitis, arthritis	DOD
29	NRAS Q61K	I/N	14	PD	35.2	-	35.2	–	NED
30	NRAS Q61K	I/N	18	PD	4.7	T	29.5	colitis	DOD
31	NRAS Q61K	N	7	CR	21.8	-	23.4	–	NED
32	NRAS Q61K	I/N	7	PD	4.2	B	18.6	–	DOD
33	NRAS Q61L	I/N	11	CR	45.4	-	45.4	colitis, hyperthyroidism	NED
34	NRAS Q61L	N	9	CR	21.0	-	21.0	–	NED
35	NRAS Q61R	P	18	PD	11.4	T	27.4	diarrhea, rash, hypopituitarism	DOD
36	NRAS Q61R	I/N	7	PD	2.9	-	8.5	rash, hypophysitis, diarrhea	DOD
37	NRAS Q61R	I/N	2	CR	24.9	-	25.5	rash, colitis, worsening RA	NED
38	NRAS Q61R	P	30	PD	9.9	T	23.6	colitis	DOD
39	NRAS Q61R	N	10	CR	20.3	-	20.3	–	NED
40	NRAS T50I, G12D	N	3	CR (TT)	44.4	T	44.4	–	NED
41	NF1 R1362	I/N	4	PD	3.2	-	5.3	colitis	DOD
42	NF1 E1734fs*4, R440*	N	3	CR	47.7	-	47.7	–	NED
43	NF1 Q1174*, E1790*	I/N	4	PD	2.0	-	2.7	diarrhea, rash	DOD
44	NF1 E1206*, Q1806*	I/N	11	CR	40.7	-	40.7	fevers	NED
45	NF1 R135W, L62*	I/N	10	CR	23.6	-	23.6	–	NED
46	NF1 splice site 5296-1G>A, splice site 6819+1G>A	N	5	CR	17.1	-	17.1	rash	NED
47	NF1 B192*	N	26	CR	52.7	-	52.7	–	NED
48	NF1 C1367*, T257fs*48	I/N	3	PD	1.8	T	2.7	hypothyroid	DOD
49	NF1 E547*, R1276Q	N	12	PD	2.3	T; B	11.1	–	DOD
50	NF1 E725fs*3, Q948*	P	9	CR	51.5	-	51.5	hypothyroidism	NED
51	NF1 G1758fs*6	N	16	CR (TT)	35.4	T	35.4	–	NED
52	NF1 K1704*	N	10	PR	8.3	-	9.3	–	DOD
53	NF1 L1906fs*13	N	12	CR	49.6	-	49.6	colitis	NED
54	NF1 loss	I/N	13	PD	13.4	T	50.3	cutaneous granulomas; flank pain, flushing, dizziness	NED

55	NF1 loss	I/N	5	PD	2.8	T; C	4.8	rash	DOD
56	NF1 loss	N	7	PD	3.0	-	23.1	hypothyroid	DOD
57	NF1 Q1055*	N	9	SD	23.0	T	23.0	–	SD
58	NF1 Q1174*, R440*	I/N	4	CR	17.2	-	17.2	pruritis	NED
59	NF1 Q1341*, R1362*, splice site 3113+1G>A	P	5	PD	5.7	-	7.9	uveitis	DOD
60	NF1 R440*	P	13	CR	17.0	-	46.5	hypothyroid	NED
61	NF1 S155fs*7	I/N	4	PD	1.4	B	3.3	–	DOD
62	NF1 S168*	I/N	19	CR (TT)	58.1	T	58.1	–	NED
63	NF1 truncation exon 10	I/N	13	PD	11.0	-	16.0	hypophysitis, elevated AST ALT, colitis	DOD
64	NF1 Y489fs*1	I/N	7	PD	2.6	-	54.4	colitis, peripheral neuropathy	NED
65	TN	I/N	24	PD	39.9	T	39.9	–	DOD
66	TN	I/N	6	PD	7.4	-	11.8	colitis	DOD
67	TN	I/N	11	CR	3.0	-	29.1	–	NED
68	TN	I/N	6	CR	24.0	-	24.0	rash, arthritis	NED
69	TN	I/N	13	PD	1.8	T; B	11.2	hypothyroidism	DOD
70	TN	I/N	4	SD	1.9	-	2.3	–	Died-other
71	TN	N	12	CR	23.7	-	23.7	–	NED
72	TN	P	42	CR	63.9	-	63.9	–	NED
73	TN	I/N	10	CR	21.8	-	21.8	rash, hypothyroidism	NED

UPN, unique patient number; BRAF F/R, BRAF gene fusion or rearrangement; TN, triple negative (no BRAF, NRAS or NF1 mutations detected); CKI, checkpoint inhibitor; I, ipilimumab; N, nivolumab, P, pembrolizumab; TT, targeted therapy; D, dabrafenib; T, trametinib; E, encorafenib; B, binimetinib; V, vemurafenib; C, cobimetinib; ORR, objective response rate; PFS, progression-free survival; OS, overall survival; OR, overall response; PD, progressive disease; CR, complete response; CR (TT), complete response due to addition of targeted therapy; SD, stable disease; PR, partial response; NED, no evidence of disease; DOD, died of disease; COD, cause of death
